# The percentage of CD133+ cells in human colorectal cancer cell lines is influenced by *Mycoplasma hyorhinis *infection

**DOI:** 10.1186/1471-2407-10-120

**Published:** 2010-03-30

**Authors:** Elisabetta Mariotti, Marica Gemei, Peppino Mirabelli, Francesca D'Alessio, Rosa Di Noto, Giuliana Fortunato, Luigi Del Vecchio

**Affiliations:** 1 CEINGE - Biotecnologie Avanzate, via Comunale Margherita 482, Napoli, 80145, Italy; 2 Dipartimento di Biochimica e Biotecnologie Mediche, Università Federico II, via Pansini 5, Napoli, 80131, Italy; 3 European School of Molecular Medicine, CEINGE - Biotecnologie Avanzate, via Comunale Margherita 482, Napoli, 80145, Italy

## Abstract

**Background:**

*Mollicutes *contamination is recognized to be a critical issue for the cultivation of continuous cell lines. In this work we characterized the effect of *Mycoplasma hyorhinis *contamination on CD133 expression in human colon cancer cell lines.

**Methods:**

MycoAlert^® ^and mycoplasma agar culture were used to detect mycoplasma contamination on GEO, SW480 and HT-29 cell lines. Restriction fragment length polymorphism assay was used to determine mycoplasma species. All cellular models were decontaminated by the use of a specific antibiotic panel (Enrofloxacin, Ciprofloxacin, BM Cyclin 1 and 2, Mycoplasma Removal Agent and MycoZap^®^). The percentage of CD133 positive cells was analyzed by flow cytometry on GEO, SW480 and HT-29 cell lines, before and after *Mycoplasma hyorhinis *eradication.

**Results:**

*Mycoplasma hyorhinis *infected colon cancer cell lines showed an increased percentage of CD133+ cells as compared to the same cell lines rendered mycoplasma-free by effective exposure to antibiotic treatment. The percentage of CD133 positive cells increased again when mycoplasma negative cells were re-infected by *Mycoplasma hyorhinis*.

**Conclusions:**

*Mycoplasma hyorhinis *infection has an important role on the quality of cultured human colon cancer cell lines giving a false positive increase of cancer stem cells fraction characterized by CD133 expression. Possible explanations are (i) the direct involvement of Mycoplasma on CD133 expression or (ii) the selective pressure on a subpopulation of cells characterized by constitutive CD133 expression.

In keeping with United Kingdom Coordinating Committee on Cancer Research (UKCCCR) guidelines, the present data indicate the mandatory prerequisite, for investigators involved in human colon cancer research area, of employing mycoplasma-free cell lines in order to avoid the production of non-reproducible or even false data.

## Background

It is well-known that mycoplasma can have adverse effects on cell cultures such as altered levels of protein and of RNA/DNA synthesis, induction of chromosomal aberrations, changes in cell membrane composition and modification of cellular morphology [1,2]. Furthermore, mycoplasma contamination can interfere with a number of biological parameters, thus influencing final data of experimental investigations [1,2].

Since human continuous cell lines derived from solid tumors are often used to explore biological and functional alterations of tumor cells, the use of mycoplasma-free cell cultures for research and specifically in isolating cancer stem cells (CSCs) is mandatory [3].

The aim of this study is to show how and to what extent the expression of CD133 antigen can be altered by *Mycoplasma hyorhinis *contamination. To address this issue, we employed the well-established GEO, SW480 and HT-29 human colon cell lines [4-6] and multidimensional flow cytometry.

As imposed by good tissue culture practice, we screened the colon cancer cell lines both for a putative *Mollicutes *contamination [7,8] and for CD133 expression [9,10]. In spite of a bright positivity for CD133 antigen during *Mycoplasma hyorhinis *contamination, after antibiotic decontamination it was possible to evidence a decrease in the percentage of CD133 positive cellular population. On these bases we decided to demonstrate that *Mollicutes *agents can have remarkable effects on the correct identification and characterization of CD133 positive CSCs subpopulation.

## Methods

### Cell Culture

Human colon cancer continuous cell lines were obtained from a research laboratory at CEINGE, Biotecnologie Avanzate (Naples, Italy). In particular, GEO, SW480 and HT-29 cell lines were cultured in DMEM, RPMI 1640 and McCoy's 5A Medium Modified (Sigma-Aldrich Corporation, St. Louis, Missouri, USA) respectively, 10% foetal bovine serum (FBS; Hyclone, Logan, UT, USA) and 1% Ultraglutamine 1 (Lonza Verviers, Belgium). All cell lines were grown in T-75 flasks (Corning Incorporated, New York) in humidified atmosphere of air containing 5% CO_2 _until complete confluence, they were then detached by trypsin/EDTA (Sigma-Aldrich Corporation, St. Louis, Missouri, USA). During the study, all cell cultures were periodically tested for the absence of bacteria and fungi contaminations by the BacT/ALERT^® ^3D (bioMérieux INDUSRTY, Hazelwood, MO, USA) instrument, an automated microbial detection system. As regards mycoplasma detection, the cell lines were cultured in absence of antibiotics for at least 2 weeks after thawing and samples were taken after a culture period of at least 2 or 7 days without medium exchange, as suggested by CC Uphoff and HG Drexler [7].

### Flow Cytometry

After enzymatic detachment from saturated cultures, GEO, SW480 and HT-29 cell lines were studied for CD133 expression (anti-CD133/1-PE; AC133 clone; Miltenyi Biotec, Auburn, CA, USA). For each sample the respective control was prepared in order to determine the level of background cellular autofluorescence without antibody staining. All samples were incubated for 20 minutes at 4°C, washed twice with PBS (Sigma-Aldrich Corporation, St. Louis, Missouri, USA) and finally analyzed using a FACSAria flow cytometer and the FACSDiva software (Becton Dickinson, Franklin Lakes, NJ). Cells were first gated on physical parameters (FSC and SSC) to exclude the majority of debris and dead cells. Afterwards, an FSC-Area *vs *FSC-Height dot plot was used to identify single cells and exclude doublets. Putative CSCs and immature cells were gated in a CD133 *vs *empty channel (FITC) dot plot.

### Mycoplasma Detection

During the study, supernatants of GEO, SW480 and HT-29 cell lines were collected and tested with the MycoAlert^® ^mycoplasma detection kit (Lonza Verviers, Belgium) using the MycoAlert^® ^assay control set (Lonza Verviers, Belgium), as suggested by the manufacturer's instructions.

All the samples were also tested by the microbiological aerobic and anaerobic agar culture.

Mycoplasma agar and broth were used as media for the detection of all mycoplasma species and were prepared according to the manufacturer's instructions (Oxoid, Hampshire, England). In particular, the mycoplasma agar was obtained by dissolving 2.84 grams of mycoplasma agar base (Oxoid, Hampshire, England) in 80 ml of distilled water and sterilized by autoclaving at 121°C for 15 minutes. After cooling, it was combined with 20 ml of mycoplasma selective supplement-G (Oxoid, Hampshire, England) and dispensed into Petri plates. In order to prepare the mycoplasma broth, 2.04 grams of mycoplasma broth base (Oxoid, Hampshire, England) were dissolved in 80 ml of distilled water and sterilized by autoclaving at 121°C for 15 minutes. After cooling, the mycoplasma broth was mixed with 20 ml of mycoplasma selective supplement-G (Oxoid, Hampshire, England) and, finally, aliquots of mycoplasma broth were imparted into tubes.

Therefore, cell culture supernatant test samples (100 μl) were pipetted onto the mycoplasma agar plates and incubated anaerobically for 14 days at 37°C. Aliquots (100 μl) of the same test sample were incubated in mycoplasma broth; after a 14 days aerobic incubation, aliquots of 100 μl were subcultured onto mycoplasma agar for 14 days in anaerobic conditions. Development of typical mycoplasma colonies, characterized by a "fried egg" appearance, was observed under the inverted microscope.

### Mycoplasma Species Determination

Mycoplasma positive GEO, SW480 and HT-29 cell lines were cultured for 7 days without antibiotics. One ml of each cell culture supernatant was centrifuged for 10 minutes at 13000 g at 4°C. The obtained pellets were washed twice with PBS (Sigma-Aldrich Corporation, St. Louis, Missouri, USA) and incubated for 15 minutes at 95°C. Mycoplasmatic DNA was extracted and purified using the Nucleon BACC2 kit (Amersham Biosciences, Freiburg, Germany). As recommended by CC Uphoff and HG Drexler [11], the PCR was performed in a final volume of 50 μl containing 100 μM of each deoxynucleotide, 1.5 μM MgCl_2_, 1.25 U Jump Start Taq DNA polymerase (Sigma-Aldrich Corporation, St. Louis, Missouri, USA) and 0,2 μM of each primer. The PCR products were subjected to the restriction fragment length polymorphism assay. In particular, the amplicons were digested for 1 hour at 37°C in a final volume of 20 μl with the restriction endonucleases AspI, XbaI, HpaII, and HaeIII (Roche Diagnostic, Mannheim, Germany). Restriction fragments were then analyzed on a 1.5% agarose gel and visualized by ethidium bromide staining. The fragments polymorphism pattern identified the mycoplasma contaminant species [11].

### Mycoplasma Decontamination

Mycoplasma eradication was accomplished by the antibiotic treatment of the infected GEO, SW480 and HT-29 cell lines. Of note, we utilized the three classes of antibiotics that were shown to be highly effective against mycoplasmas [12]. In fact, quinolones inhibit DNA replication, whereas tetracyclines and macrolides hamper protein synthesis [12].

In particular, infected cells were treated with the quinolones enrofloxacin (Fluka, BioChemika, distributed by Sigma-Aldrich Corporation, St. Louis, Missouri, USA) and Mycoplasma Removal Agent (Euroclone, Lugano, Switzerland) at concentration of 25 μg/ml and 0.5 μg/ml, respectively, for 7 days. Cells were also exposed to the quinolone ciprofloxacin (Fluka, BioChemika, distributed by Sigma-Aldrich Corporation, St. Louis, Missouri, USA) at the concentration of 10 μg/ml for 14 days. Moreover, the anti-micoplasmatic treatment, included the BM Cyclin 1 (Roche, Mannheim, Germany), containing the macrolide tiamulin, for 3 days at the final concentration of 10 μg/ml, followed by the BM Cyclin 2 (Roche, Mannheim, Germany), containing the tetracycline minocycline, for 4 days at the concentration of 5 μg/ml.

Finally, MycoZap^® ^mycoplasma elimination reagent (Lonza Verviers, Belgium) was used. The kit includes a combination of antibiotic and antimetabolic agents protected by a patent. MycoZap^® ^reagent 1 was used for 4 days, while reagent 2 was used for 12 days, according to the manufacturer's data sheet.

As for mycoplasma detection, all antibiotic-treated cell lines were cultured for at least 4 weeks without any chemotherapic molecules.

### Co-cultivation of Mycoplasma with Cell Cultures

Approximately 80% confluent monolayer of mycoplasma-eradicated GEO cell line was used for the *Mycoplasma hyorhinis*-driven infection. The growth of the *Mycoplasma hyorhinis *was carried out using the media and culture conditions recommended by the supplier (ATCC, Rockville, MD).

On the day of infection, the old medium was replaced with fresh medium containing the dilution of viable mycoplasma at a concentration of about 10 CFU/ml, previously determined by the microbiological aerobic and anaerobic agar culture. The infected cells were then incubated at 37°C, 5% CO_2_. Every 7 days, supernatants were tested for mycoplasma detection and, in case of positive result the cells were detached with trypsin/EDTA and processed for cytometric analysis.

## Results and Discussion

*Mollicutes *contamination is recognized to be a critical issue for the cultivation of continuous cell lines and about 30% of all cellular models are reported as mycoplasma-infected with a limited number of human species origin [11]. It is well-known that *Mollicutes *agents can heavily interfere with cellular activities such as growth rate, DNA, RNA and protein synthesis as well as cause significant cell morphology alterations [13-16]. Considering that one of the main objectives of our research project was the characterization and isolation of CD133-positive CSCs in human cellular models, we carefully checked the employed cell lines to detect *Mollicutes *agent contamination as indicated by good tissue culture practice.

**Figure 1:**
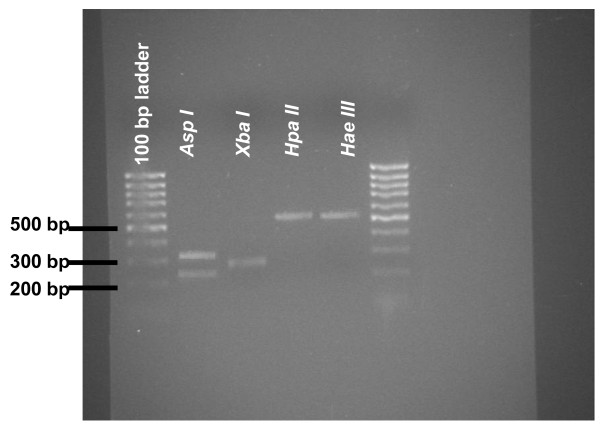
**Determination of the mycoplasma species by restriction fragment length polymorphisms analysis of PCR products**. The fragment pattern identifies the contaminant as *Mycoplasma hyorhinis*.

MycoAlert^® ^assay [8] showed that GEO, HT-29 and SW480 cell lines were positive to *Mollicutes *agent contamination, being the B/A score of 62, 53 and 16, respectively (data not shown). Later on, these results were confirmed by aerobic and anaerobic microbiological agar culture (data not shown). In order to determine the contaminant species, we performed the analysis of modified restriction fragment length polymorphisms. The application of this method revealed that GEO, SW480 and HT-29 cell lines were infected by *Mycoplasma hyorhinis*, one of the most common cell culture contaminants encountered [17] (Figure 1). Although in the past, this cell culture contaminant was thought to derive from bovine sera, nowadays FBS is tested for mycoplasma contamination and therefore microrganisms are more likely to spread from one cell culture to another by inadequate cell culture techniques [17].

**Figure 2:**
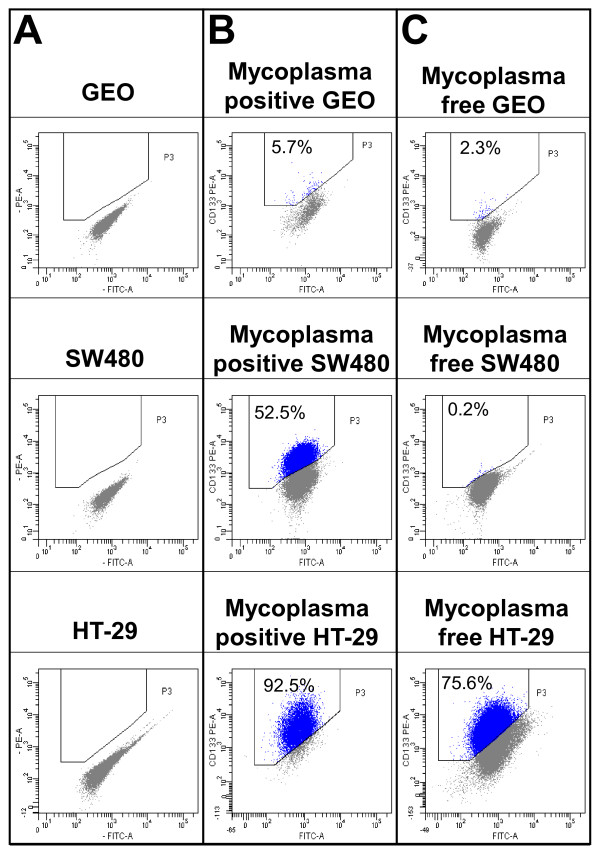
**Cytometric analysis of CD133 expression in GEO, SW480 and HT-29 cell lines**. Panel A shows control cells without antibody. Panel B shows *Mycoplasma hyorhinis*-positive cells. Panel C shows mycoplasma-free cells.

Cell lines for CD133 expression were then characterized (Figure 2). A negative control was prepared for each sample in order to determine the level of background cellular autofluorescence without antibody staining (Figure 2, Panel A). We observed the clear-cut separation of a CD133+ population, that was equal to 5.7%, 52.5% and 92.5% in case of GEO, SW480 and HT-29 Mycoplasma contaminated cell lines, respectively (Figure 2, Panel B).

All cell lines underwent the eradication protocol and were successfully decontaminated by BM Cyclin 1 and 2 treatment. After the combined use of MycoAlert^® ^and microbiological agar culture detection assay showing the absence of the contaminant, the three cell lines for CD133 expression were screened in a new cytometric set of experiments. As shown in Figure 2, Panel C, a remarkable CD133 expression decrease was observed resulting in 2.3%, 0.2% and 75.6% in case of GEO, SW480 and HT29, respectively.

**Figure 3:**
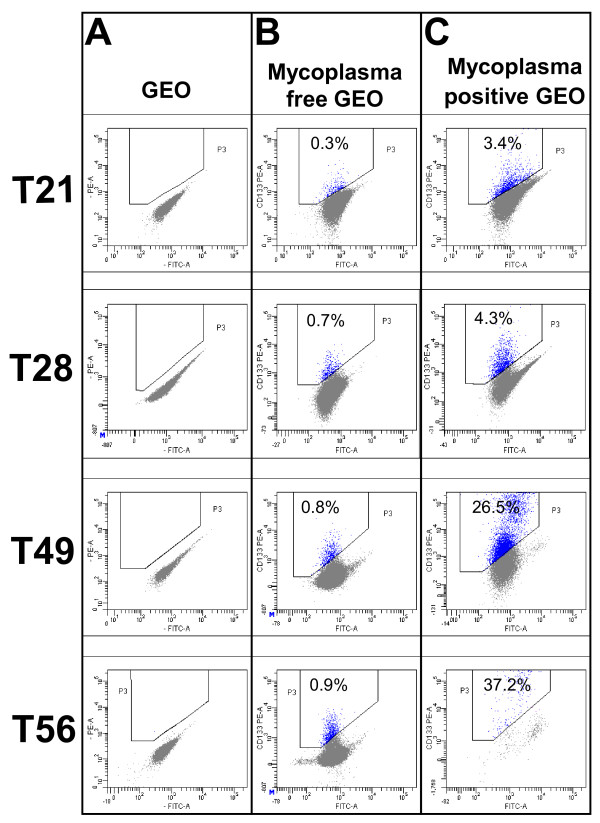
**Cytometric analysis of CD133 expression in GEO at different post-infection time**. Panel A shows GEO cells without antibody. Panel B shows mycoplasma-free GEO cells. Panel C shows *Mycoplasma hyorhinis*-positive GEO cells.

In order to confirm the ability of *Mycoplasma hyorhinis *to increase the percentage of CD133+ cells in human colon cancer cell lines, we re-infected GEO cells and checked the cell culture supernatant by MycoAlert^® ^analysis once a week. The results of this experiment showed that *Mycoplasma hyorhinis *was detectable only after 21 days of co-incubation with GEO cells at 37°C and 5%CO_2_. For each step of the experiment, the respective negative control was prepared in order to determine the level of background cellular autofluorescence without antibody staining (Figure 3, panel A). As shown in panel B and C of Figure 3, cytometric analysis demonstrated a significant (P = 0,0286, Mann-Whitney test) increase of CD133+ population in *Mycoplasma hyorhinis *positive GEO cells compared to the same microorganism-free GEO control cells. In fact, 3.4%, 4.3%, 26.5% and 37.2% CD133+ cells were detected on days 21, 28, 49 and 56 post infection respectively, whereas 0.3%, 0.7%, 0.8% and 0.9% were found on days 21, 28, 49 and 56 respectively, in mycoplasma-free control cells.

## Conclusions

Our results demonstrate that *Mycoplasma hyorhinis *infection can play an important role on cultured human colon cancer cell lines giving rise to considerable false positive increase of CSCs cellular fraction characterized by CD133 expression. This occurrence may be explained by: i) the direct influence of mycoplasma on anti-CD133 MoAb binding sites or ii) the ability of CD133 positive cells to survive despite the toxic effects exerted by the mycoplasma.

In conclusion, methods for the detection, elimination and prevention of *Mollicutes *contamination should be included in the basic panel of CSCs research techniques. We are confident that technical advancements in cell lines handling will facilitate CSCs studies, positively influencing oncological research improvement in the near future.

## Competing interests

The authors declare that they have no competing interests.

## Authors' contributions

EM conceived the study and performed the cell culture experiments. MG performed flow cytometry experiments. PM and FDA contributed to data analysis. RDN analyzed data and wrote the manuscript. GF participated in data interpretation and revision of the manuscript. LDV conceived the study, designed the experiments and wrote the manuscript together with RDN. All authors read and approved the final manuscript.

## Pre-publication history

The pre-publication history for this paper can be accessed here:

http://www.biomedcentral.com/1471-2407/10/120/prepub
